# Multimodal Sensing Capabilities for the Detection of Shunt Failure

**DOI:** 10.3390/s21051747

**Published:** 2021-03-03

**Authors:** Milenka Gamero, Woo Seok Kim, Sungcheol Hong, Daniel Vorobiev, Clinton D. Morgan, Sung Il Park

**Affiliations:** 1 Department of Electrical and Computer Engineering, Texas A&M University, College Station, TX 77843, USA; mgamero@tamu.edu (M.G.); wooseok.kim@tamu.edu (W.S.K.); hyhaerong@tamu.edu (S.H.); dansparrow99@tamu.edu (D.V.); 2 Department of Neurosurgery, Barrow Neurological Institute, Phoenix, AZ 85013, USA; Clinton.Morgan@BarrowBrainAndSpine.com; 3 Center of Remote Health Sciences and Technologies, Texas A&M University, College Station, TX 77843, USA; 4 Institute for Neuroscience, Texas A&M University, College Station, TX 77843, USA

**Keywords:** shunt device, multimodal sensors, pressure and flow rate

## Abstract

Hydrocephalus is a medical condition characterized by the abnormal accumulation of cerebrospinal fluid (CSF) within the cavities of the brain called ventricles. It frequently follows pediatric and adult congenital malformations, stroke, meningitis, aneurysmal rupture, brain tumors, and traumatic brain injury. CSF diversion devices, or shunts, have become the primary therapy for hydrocephalus treatment for nearly 60 years. However, routine treatment complications associated with a shunt device are infection, obstruction, and over drainage. Although some (regrettably, the minority) patients with shunts can go for years without complications, even those lucky few may potentially experience one shunt malfunction; a shunt complication can require emergency intervention. Here, we present a soft, wireless device that monitors distal terminal fluid flow and transmits measurements to a smartphone via a low-power Bluetooth communication when requested. The proposed multimodal sensing device enabled by flow sensors, for measurements of flow rate and electrodes for measurements of resistance in a fluidic chamber, allows precision measurement of CSF flow rate over a long time and under any circumstances caused by unexpected or abnormal events. A universal design compatible with any modern commercial spinal fluid shunt system would enable the widespread use of this technology.

## 1. Introduction

As life expectancy and demand for excellent medical healthcare increase, there is greater demand for innovative bioelectronic devices that can continuously monitor and provide reliable real-time information for an accurate diagnosis, as well as assuring correct functionality of the implantable device in different fields such as cardiovascular, pathological and neurological diseases [1,2,3,4]. Hydrocephalus is a neurological disorder in which there is an excess of cerebrospinal fluid (CSF) within the brain, and may develop as a consequence of trauma, infection, tumors, intracranial hemorrhage, or congenital defects. In the United States, one in every 770 babies has hydrocephalus—a complex, life-threatening condition marked by an accumulation of CSF within the brain [5,6,7,8,9]. Depending on its severity and duration, hydrocephalus may cause progressive neurological decline, blindness, impaired motor function, urinary incontinence, dementia, or death. A shunt system is the mainstay of long-term management [10,11,12], consisting of a proximal in-catheter, a valve, and a distal out-catheter. The system diverts CSF from the ventricles within the brain, or the subarachnoid spaces around the brain and spinal cord, to another body region where it will be absorbed. This creates an alternative route for removal of the accretion of CSF within the brain, and usually restores the physiological balance between the build-up, flow, and absorption of CSF when one or more of these functions are impaired. Shunts have been the primary treatment for hydrocephalus for nearly 60 years [13,14,15]. However, shunt failures and concerns are extremely common matters and a significant burden on the healthcare system. The crucial issues of long-term management of patients can often occur in silos, where managing providers have insufficient information about critical questions, such as original shunt indications, where the shunt was placed, the shunt function, the stray at proximal or distal retained catheters, the history of valve setting changes, or typical symptoms during shunt failure.

Recent surveys on the causes of shunt failure are steadily emerging and commonly mention several issues, such as obstruction, mechanical failure, over drainage, loculation, and abdominal complications of the shunt [16,17,18]. In addition, there have been attempts to apply other options while using differential pressure as the priority predictor, but the effect on obstruction, the main cause of shunt failure, is insignificant [19]. Despite developments in novel shunt technology and shunt failure prevention research, there has been minimal technological advancement in the field [20,21]. A recent study proposed to determine whether a shunt is malfunctioning through a non-invasive device that can estimate the catheter flow rate as a function of temperature change. The existing non-invasive approach uses localized thermal actuation, but CSF flow rate calculation is heavily dependent on variable parameters, such as skin thickness and thermal properties that vary in individuals [22,23,24,25,26,27,28,29,30]. This indicates that a threshold for detection of shunt failure can vary in individuals, and that a setting for measurements of CSF flow rate must be customized to an individual. It can also change in the same individual at different times and under different circumstances. For example, displacement of a skin-mounted device or detachment from the skin due to contraction of adjacent muscle tissues, movement of a subject, or releasement of sweat during measurements, can significantly affect measurements and degrade signal integrity. Our system, in contrast, is independent of the position of the catheter with respect to the surface of the skin.

Here, we present a multimodal sensing wireless electronic device that would permit minimally invasive measurements of flow rates of CSF via flow sensors and electrodes embedded in a fluidic chamber and transmit data to your smartphone. It consists of a flow sensor, electrodes, a fluidic chamber, and a wireless communication module. All of these components are mounted on a flexible substrate, such as polyimide, and the whole system is encapsulated by soft polymers, such as polydimethylsiloxane (PDMS). The device that is attached in-line with a shunt device would enable unique wireless telemetry to monitor flow rates using two different factors: pressure and resistance in shunt systems with high accuracy [31]. Such a novel shunt technology would allow patients with hydrocephalus to have a highly reliable, non-invasive mechanism for shunt failure detection with a device adaptable to multiple different shunt valve manufacturers.

## 2. Materials and Methods

### 2.1. Pattern Fabrication

A copper (Cu, thickness: 18 µm)/polyimide (PI, thickness: 12 µm) bilayer film (AC181200R, DuPont™ Pyralux^®^, Wilmington, DE, USA) was placed on a glass slide (dimension: 2” × 3”). Next, 2.5 µm photoresist (AZ^®^1518, AZ Electronic Materials, Somerville, NJ, USA) was deposited on top of the copper bilayer film through spin-coating at 3000 r.p.m. for 30 s and baked on a hot plate at 100 °C for 4 min. Then, UV photolithography with UV intensity for 265 mJ/cm^2^ defined patterns on the prepared film. In the last step, the procedures of solvent development (AZ^®^Developer 1:1, AZ Electronic Materials, Somerville, NJ, USA) for 30 sec, and wet etching (copper etchant, Alfa Aesar™, Tewksbury, MA, USA) for 3 min, formed patterns, including copper traces and contact pads, on a flexible substrate.

### 2.2. Fabrication of Fluidic Chamber

The shunt chamber was produced in a three-dimensional printer (Form 2, Formlabs Inc., Somerville, MA, USA) with material FLGPCL04 (Young Modulus (E) 1.6 GPa). Then, it was washed with isopropyl alcohol (IPA) for 15 min and, finally, cured by the heating system (Form Cure, Formlabs Inc., Somerville, MA, USA) at 60 °C for 30 min.

### 2.3. Device Assembly and Encapsulation

All components, including the Bluetooth chip (nRF52832, Nordic semiconductor, Trondheim, Norway), battery, transistors, a reed switch, resistors, and capacitors, were mounted on a flexible PI/Cu (thickness: 30 µm) substrate. The pressure sensor with a range of gauge pressure 0–1 psi (MPRLS0001PG0000SA, Honeywell, Charlotte, NC, USA) and two wire-filaments were inserted through holes in the shunt chamber. Application of transparent silicone sealed the holes in the shunt chamber. Next, biocompatible polymers, PDMS (SylgardTM 184 silicone elastomer kit, Dow^®^, Midland, MI, USA), encapsulated all components of the device by a simple dipping process, and the device was cured in a vacuum oven at 80 °C for 1 h. These processes yielded a wireless multimodal sensing device.

### 2.4. Finite Element Method Simulations

We used a commercial finite element analysis tool, Abaqus/CAE 2016 (Dassault Systemes, Vélizy-Villacoublay, France), with the package CFD (Computational Fluid Dynamics), to obtain pressure variations along with the shunt chamber in different flow rates. Viscosity and density parameters used for CSF simulations were 0.001 Pa·s and 1 g/mL, respectively. A catheter with a length of 45 cm connected the syringe pump with the inlet of the shunt chamber, and a catheter with a length of 35 cm connected the outlet of the shunt chamber with the reservoir. We used the equation:

v = Q/A,(1)

where Q—flow rate; A—cross-sectional area as an input to the syringe pump. We performed simulations using the above parameters to obtain the pressure in the shunt chamber under conditions of different diameters (1.0/1.1/1.2/1.3/1.4 mm) and different flow rates (0.01/0.1/0.3 mL/min).

### 2.5. Experimental Assays

Experimental assays consisted of a syringe pump (Fusion 100, Chemyx Inc., Stafford, TX, USA), catheters, and a multimodal sensing device with a fluidic chamber embedded. Here, the syringe pump controlled flow rates from 0.3 to 0.01 mL/min. We tested three different flow rates: 0.01, 0.1, and 0.3 mL/min. Here, a flow rate of 0.3 mL/min indicated a shunt was operating normally, 0.01 mL/min represented a shunt failure associated with obstructions in a catheter [23,24,32], and 0.1 mL/min was chosen as a mid-point to evaluate the changes in pressure and resistance. We monitored pressure and resistance over 30 min at various flow rates under two different conditions: dry and wet conditions. The dry condition indicated the distal end of a fluidic chamber was exposed to air, while the distal end of it was immersed in a reservoir in the wet condition.

### 2.6. Data Acquisition

The device employed an actuation mechanism by which a magnetic field, here a magnet, triggered a reed switch, which was part of the switching circuit (Figure S1). The reed switch closed (or was activated) in response to a magnetic field [33]. The data communication between the Bluetooth chip and the pressure sensor occurred via Serial Peripheral Interface (SPI) protocol. The Bluetooth chip also monitored the resistance of fluids contained in a fluidic chamber via embedded electrodes. The Bluetooth chip integrated measurements of flow rates with two sensors: pressure and resistance. The pressure was measured through the sensor MPRLS0001PG0000SA-Honeywell. As our device will be placed in an abdomen cavity and monitor the differentials in pressures at the distal end of a catheter, the pressure sensor was chosen considering intra-abdominal pressure (IAP). IAP ranges from 5 to 7 mmHg (0.09–0.13 psi) in healthy patients and up to 0.27 psi for obese patients [34]. The pressure sensor selected can detect values from 0 to 1 psi. For resistance measurements, the diameter conduit of the shunt should be large enough to avoid blockage. Usually, the conduits of the shunt are not completely filled with CSF [35,36,37]. The fluid volume flowing through our device was directly related to the CSF flow rate and would be scaled by resistance measured by two electrodes located near each inlet or outlet. A decrease in the fluid volume due to obstruction of the catheters leads to an increase in resistance. If the flow rate is reduced further, to the point where there is no noticeable or detectable change (a flow rate of 0.01 mL/min), this will leave a chamber empty, or a small portion of the chamber would be filled with fluids. As such, high resistance over 10 MΩ was measured between the two electrodes. Here, a resistor of 10 MΩ with fluid resistance connected in series constructs a bridge configuration for measurements, and any variation in resistance would then be monitored by the Bluetooth chip. Measurement of pressure and resistance is sampled at 1 Hz and sent to a smart device via Bluetooth protocol. A portable device displays measurements on the screen in real-time. A user can retrieve data from the mobile device and analyze it when necessary.

## 3. Results

**Figure 1:**
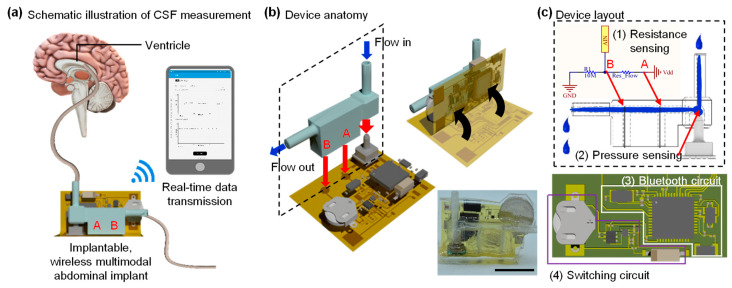
Overview of multimodal sensing device for the detection of shunt failure. (**a**) Schematic illustration of the proposed platform, including a multimodal sensing device composed of pressure, resistance measurement and portable device that can display measured data in real-time. (**b**) Illustration of device anatomy and procedures for device fabrication. Image of a multimodal sensing device (bottom right); the scale bar is 10 mm. (**c**) A layout of fluidic chamber that can measure two different types of data for precision measurements of flow rate (top), flexible printed circuit board with components that include the Bluetooth circuit and the switching circuit for turning the device ON/OFF (bottom).

Procedures for real-time measurements of CSF appear in Figure 1a. Here, a catheter from the brain (or a syringe pump) connected to the input of the shunt chamber, and fluids in the chamber drained out the abdomen cavity (or a reservoir). A Bluetooth wireless system monitors flow rates via multimodal sensors that consist of a pressure sensor and electrodes embedded in the chamber. The system transmits measurements to a smartphone and/or a portable device, which can display the received measurement data on the screen in real-time. Illustrations of the fabrication processes, device anatomy, and layouts are in Figure 1b,c.

### 3.1. Chamber Design Decision Based on Simulation Results

**Figure 2:**
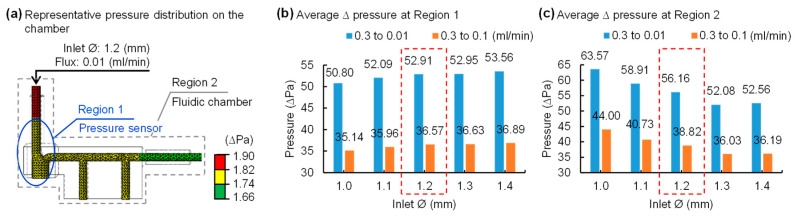
Finite element method analysis of pressure distributions on a fluidic chamber. (**a**) Representative pressure distribution on the fluidic chamber with an inlet diameter of 1.2 mm at 0.01 mL/min flow rate. Region 1 (blue) indicates the zone nearby pressure sensor vertically and region 2 (gray) corresponds to the whole fluidic chamber. Plots of pressure vs. diameter of inlet at region 1 (**b**) and region 2 (**c**), respectively. The average values of pressure difference according to the reductions in flow rates show opposite results in region 1, increasing as the inlet diameter increases, and, in region 2, decreasing as the inlet diameter increases.

Figure 2a shows a representative pressure distribution result among a variety of simulations in the proposed fluidic shunt chamber design. The geometry of the chamber, and the locations of an inlet and outlet, could affect the flow rate because fluids flow through the chamber. Therefore, the determination of structure dimensions, in particular the diameter of an inlet and outlet of a chamber, is critical for the realization of robust, precision measurements of flow rates. In this study, we performed finite element analysis using a commercially available tool, Abaqus, to determine the dimensions of a fluidic chamber that lead to the maximum change in flow rates. We obtained pressure distributions in the shunt chamber under conditions of the different flux 0.01/0.1/0.3 mL/min with various input diameters values from 1.0 to 1.4 mm with a 0.1 mm interval. Here, atmospheric pressure was considered at the end of the output catheter and set to zero. The pressure variable was enabled to measure at each location: region 1 and 2 (Figure 2a). The simulation results revealed that the differential pressure increases as the inlet diameter of the chamber increases in region 1 (Figure 2b), whereas the opposite trend is apparent in region 2 (Figure 2c). Since the proposed device aims for more precise and minute measurements than conventional methods, we desire results that are not biased to one side of the partial or whole fluidic chamber. Collectively, considering the overall simulation analysis, plus the pressure distribution around the pressure sensor and the entire region, a chamber with an inlet diameter of 1.2 mm is an optimal setting for accurately measuring pressure changes.

### 3.2. A Fine Multimodal Sensor Capable of Measuring Minute Changes in Flow Rates

**Figure 3:**
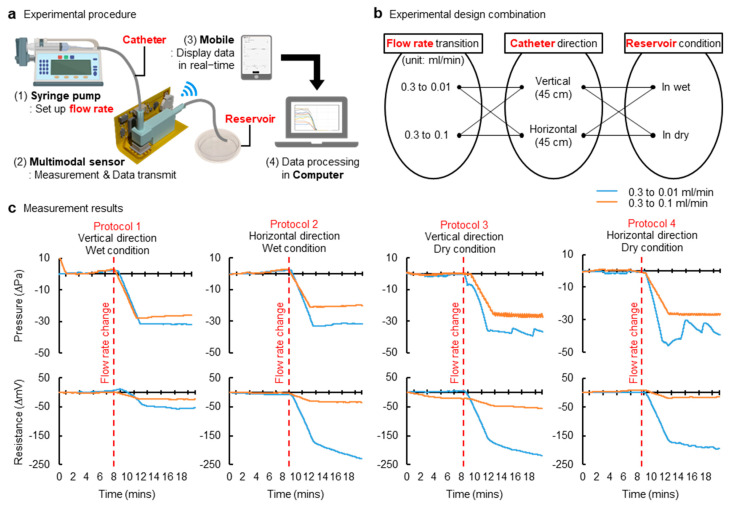
Measurement results in various conditions. (**a**) Schematic illustration of a measurement setting. (**b**) Summary of various configurations. Each configuration is characterized by a flow rate transition, catheter direction, and reservoir condition. Two types of flow rate transition: when the initial flow rate is 0.3 mL/min, and then, it changes to 0.01 or 0.1 mL/min. Catheter direction, vertical and horizontal, represents the upright and supine anatomical position, respectively. The reservoir condition emulates the abdomen cavity or other area where the cerebrospinal fluid (CSF) is drained. Dry condition is when the external pressure is the atmospheric pressure, and wet condition is where the reservoir is filled with fluids. (**c**) Plots of pressure and resistance measurements at various configurations described in (**b**). Protocol 1 is the vertical direction and wet condition. Protocol 2 is the horizontal direction and wet condition. Protocol 3 is the vertical direction and dry condition, and Protocol 4 is the horizontal direction and wet condition. These four protocols are performed under conditions where the initial flow rate is 0.3 mL/min, and then it changes to 0.1 or 0.01 mL/min.

We fabricated a shunt chamber and a multimodal sensing device using parameters found in the results from simulations (Figure 2). Figure 3a shows a measurement setup, and protocols for experiments appear in Figure 3b. Here, the system was tested in each setting: the vertical and horizontal setting (Video S1). Each setup represented a patient standing up and laying down, respectively. In each mode, we measured pressure and resistance at minute changes of various flow rates under both wet and dry conditions, considering the shunt chamber outlet is exposed to air or immersed in solutions; the results are summarized in Figure 3c. Experimental measurement results revealed that differential pressures stayed in phase with that of resistances in different flow rates of any of the conditions listed above. Moreover, the results implied that the proposed multimodal sensing mechanism improves accuracy, since it includes a dual-sensing system and thereby ensures a robust, reliable diagnosis of flow rate, though even this changed slightly. An anatomical position—such as supine or upright position—and pressure at the outlet of the distal catheter can influence pressure measurement, and, under this circumstance, a variation in flow rates would be marginal or negligible. This may guide us to misleading flow rates. This suggests that a single modality (measurements of pressure) may not be sensitive and/or accurate enough to characterize flow variation and anatomical position condition. However, multimodality, presented in this study as the combination of measurements of pressure and resistance, can help to avoid false detection of flow rates and therefore improve accuracy. For example, a change in flow rates due to catheters’ obstruction would also affect a fluidic chamber at the distal end and would leave a chamber empty or partially filled with fluids, even if pressure is marginal or not big enough for detection. This will ensure against false detection of flow rate associated with improper positioning of a subject and thereby maintain signal fidelity. This is a way to characterize the flow rate variation without being misled by the anatomical position and/or the pressure at the outlet of the distal catheter.

Offering a highly sensitive flow sensor is critical because even the ordinary CSF flow rate is low. Unfortunately, this fact increases the likelihood that a deceptive or erroneous report, associated with an abrupt change due to a patient’s natural motion during measurements, can be made, and it could degrade signal fidelity. Consequently, the proposed multimodal sensing mechanism can mitigate the risk linked in a scenario described above.

### 3.3. Energy-Efficient Wireless Telemetry

**Figure 4:**
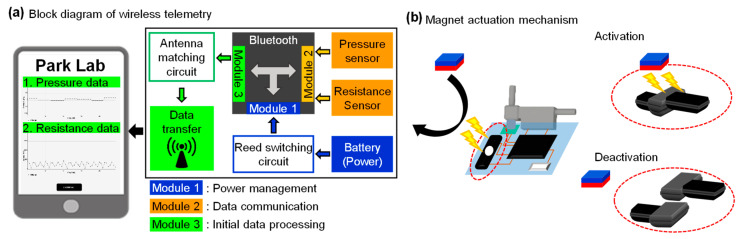
Overview of the proposed wireless telemetry. (**a**) Functional block diagram of a multimodal sensing device and a portable device. (**b**) Illustration of an actuating mechanism for switching enabled by a reed switch embedded device and a bar magnet.

**Figure 5:**
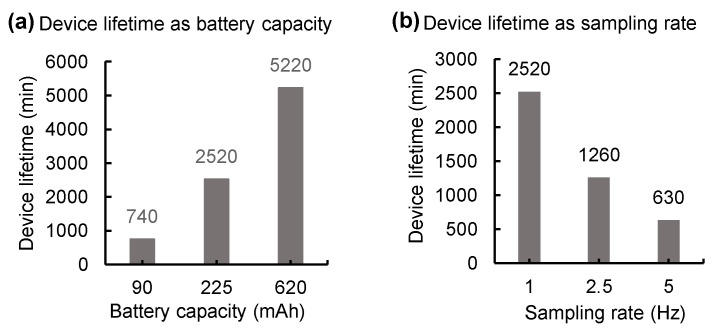
Measurements of device lifetime. (**a**) A plot of device lifetime vs. battery capacity. Here, a device samples at a rate of 1 Hz. (**b**) A plot of device lifetime vs. sampling rate; a 225 mAh battery capacity used for measurements.

Figure 4a shows a functional block diagram of the proposed wireless telemetry, which provides a user-friendly interface of the portable device that can display real-time measurement data of pressure and resistance transmitted from a multimodal sensing device. We provide two user interfaces for convenience: one is a mode that allows users to check all the data in detail, as shown in Figure 4a and video S1, while the other guards noteworthy values in allowing users to readily detect changes (Figure S2). This wireless device employs an actuation mechanism enabled by a magnet and offers a distinct operation mode; a reed switch embedded in the device is activated when a bar magnet is placed in proximity to the wireless device. This device can be deactivated by the same procedure described above (Figure 4b). Such an energy-saving operation mode allows for the extension of battery life. Measurements of battery lifetime appear in Figure 5. The results demonstrated that 2 min daily usage of the device with a 620 mAh battery capacity and 1 Hz sampling rate guarantees operation for seven years without recharging it. We used three different sampling rates, 1, 2.5, and 5 Hz, for assessment of power consumption and detection accuracy. Results revealed that a device at each sample rate consumes 5.3, 10.7 and 21.4 mA at 3 V, respectively, and that the performance was optimized at a sample rate of 1 Hz. Compared with other approaches in Krishnan et al. (6 mA during operation with 5 Hz) and Jetzki et al. (6 mA at 3.6 V), our device requires less power or current for operation [22,38].

## 4. Discussion

### 4.1. Encapsulation for Long-Term Operation

The proposed wireless device will be exposed to biofluids when implanted in a human, and must maintain functionality in such an environment over a significant period [39,40]. The device is currently encapsulated within soft polymers, such as PDMS. We performed durability tests with devices (*n* = 8) where they are immersed in saline solution and monitored at temperatures 27 and 50 °C; measurements are found in Figure S3. The results revealed that a device maintains functionalities in a wet condition over two months. However, soft encapsulation materials do not provide levels of isolation from biofluids over a year [41]. One way to achieve similar levels of isolation is to use titanium or ceramic packages. Titanium encasement can serve as a long-term barrier to biofluids; however, such metal housing interferes with ultra-high frequency (UHF) communications between an implanted wireless device and a portable device and degrades signal integrity. That said, near field communication (NFC) can be an alternative because it is almost insensitive to surrounding metals at frequency ranges from 300 kHz to 30 MHz. Furthermore, a ceramic package can be combined with a UHF or NFC platform due to the nature of transparency to all electromagnetic radiations.

### 4.2. Battery-Free Operation

The actuating mechanism enabled by a reed switch can extend battery life to up to seven years. Radiofrequency (RF) energy harvesting could be another embodiment of the proposed approach [39,42,43,44]. A wireless coil embedded in an implanted device induces inductive coupling between an implanted device and a portable device to power the wireless Bluetooth or NFC chip. Such battery-free operation eliminates the need for a large battery pack, allowing miniaturized construction of the device and therefore permitting low-cost construction and low maintenance. The nature of this approach reduces power consumption down to mW level, and thereby provides more advantages in power budgets while minimizing the side effects associated with tissue damage caused by heat generation from a power harvesting unit. It can also provide access to an embedded memory in which critical information, such as historical flow rate data and shunt setting history, can be stored. Given these possibilities, the Bluetooth chip we are applying offers a high level of security clearance and facilitates reliable read/write access on portable devices, which is advantageous for extending the mentioned functionality [45,46].

### 4.3. Functional Expansion for Multi-Sensing

The novel shunt device can be extended to include capability for multimodal sensing variations of flow rates of CSF. There are various methods to calibrate CSF flows: flow sensor, strain gauge, pressure sensor, thermal actuator, and direct resistance measurements of fluids. A combination of two or more modalities would suggest exceptional accuracy and provide zero risks for misdiagnosis. Furthermore, combined modalities allow one to identify a new signature for the malfunction of a shunt device.

## 5. Conclusions

The proposed study will cite the potential for significant human benefits. First, a novel multimodal sensing wireless system for shunt technology would dramatically improve the quality of life of people with hydrocephalus by providing strategies for managing medical uncertainty. A real-time update of information from the proposed wireless system will empower patients with a shunt to diagnose shunt failure. Second, it would reduce the need for additional surgeries when shunt malfunctions happen. Collectively, the proposed multimodal sensing wireless system for shunt technology would enable the extension of these successes to devise new ways to improve shunt failure detection.

## 6. Patents

The subject matter of the manuscript is protected by a PCT application (US Appl. 62/719, 398, filed 17 August 2020). This PCT application has been nationalized in multiple jurisdictions, including the United States and European Union.

## Data Availability

The data that support the plots within this paper and other finding in this study are available from the corresponding author upon reasonable request.

## Supplementary Materials

The following are available online at https://www.mdpi.com/1424-8220/21/5/1747/s1, Figure S1: Switching circuit, Figure S2: A screenshot of the user-friendly interface of the portable device, Figure S3: Characteristics of the durability of devices at room temperature and 50 °C and Video S1: Demonstration of measurement of flow rates in a vertical setting.
