# Supplementary material for: Multimodal Sensing Capabilities for the Detection of Shunt Failure
Source: Sensors (Basel). 2021 Mar 3;21(5):1747. doi: 10.3390/s21051747 (PMC7959456; doi:10.3390/s21051747)
Supplement: Supplementary file 1 [file sensors-21-01747-s001.zip › Supplementary Figures_rev1.pdf]

Figure S1

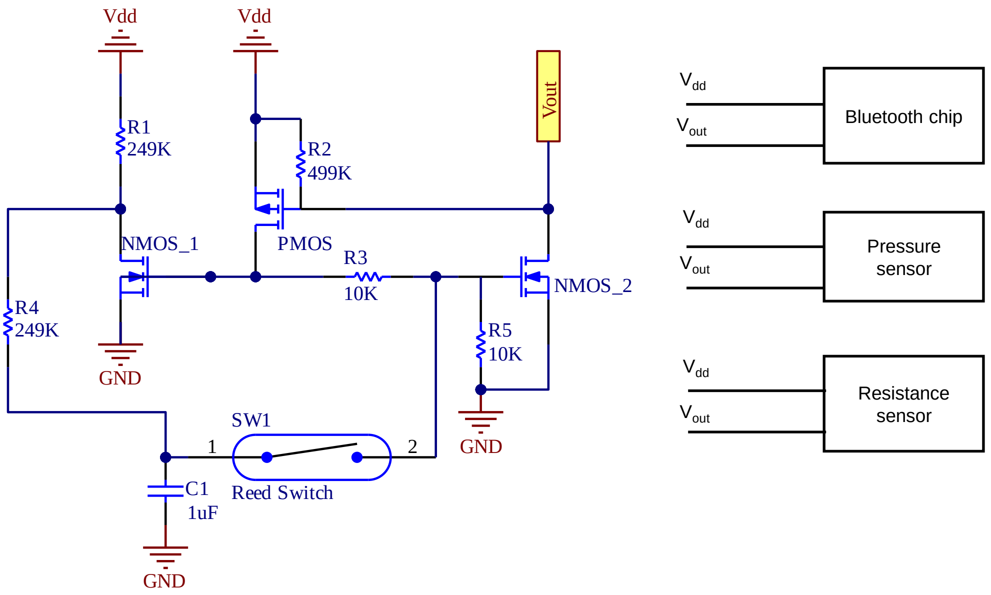

Figure S1. Switching circuit. Here, Vout serves as a node that powers the Bluetooth module, the pressure, and the sensor. The system is turned ON when a voltage level at Vout is low (or OFF when Vout is high). The reed switch (SW1) is normally opened, and it is closed in response to a magnetic field. Initially, the capacitor C1 is being charged through R1 and R4, and a voltage level at Vout remains high. When SW1 closes, C1 is discharged through R5, and NMOS_2 is activated. This causes the voltage at Vout to be zero (or low), and subsequently, PMOS and NMOS_1 are activated. As a result, currents flow through NMOS_1 while avoiding C1 to be charged, and the rest of the circuitry is powered. After SW1 restores to the normal state, NMOS_2 remains activated. This actuation mechanism allows for reverse operation. If the reed switch is activated again by a magnet, NMOS_2 becomes deactivated. A voltage level at Vout is back to high. As a result, PMOS and NMOS_1 become deactivated, allowing C1 to be charged. So, the pathway between the Bluetooth module and the battery is shut down. For antenna matching, we used a configuration recommended in the manual for the Bluetooth nRF52832 chip Nordic semiconductor (nRF52832 Product Specification v1.4). The antenna operates at a frequency of 2.4 GHz, and the matching circuit consists of an inductor (3.9 nH) and a capacitor (0.8 pF).

Figure S2

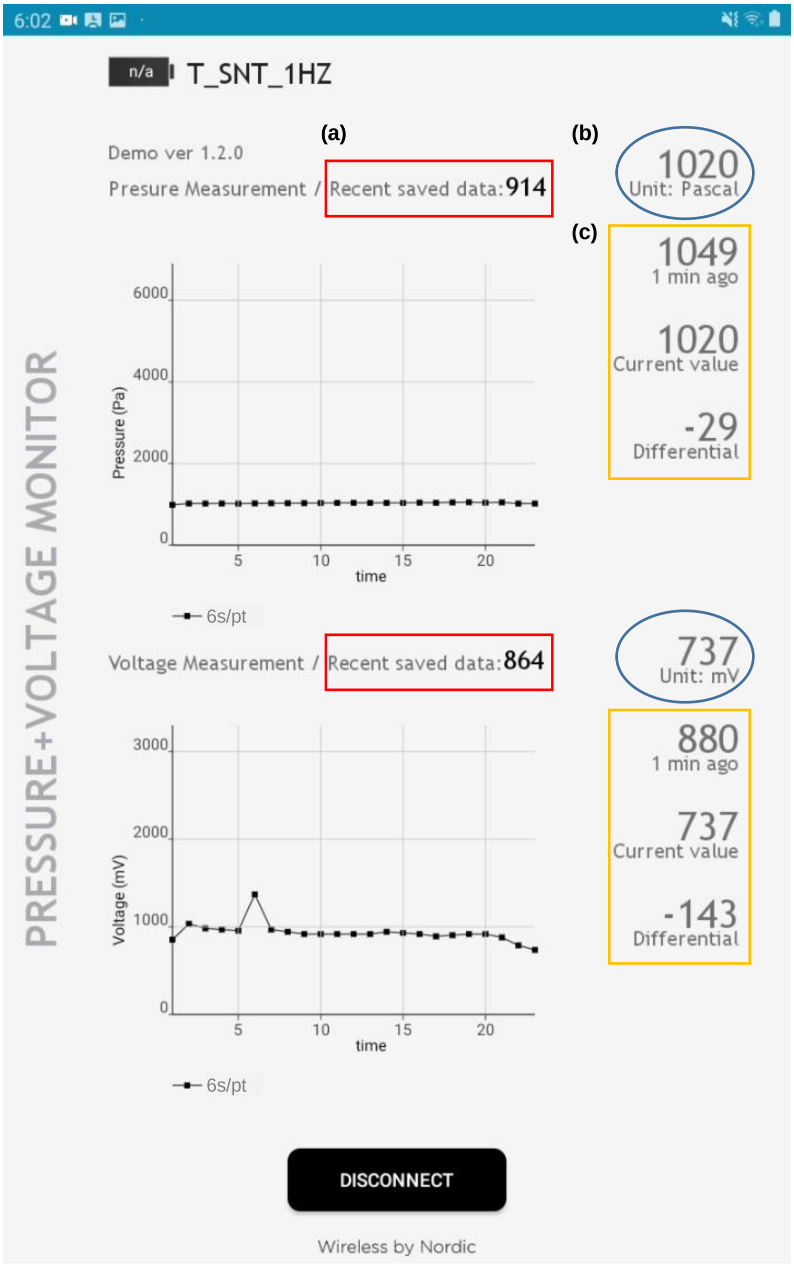

Figure S2. A screenshot of the user-friendly interface of the portable device. (a) Loaded the last value of the recent operation. (b) Refreshed the currently measuring value at every six seconds. (c) Every minute, these values were refreshed. Specifically, if the both "Differential" values show negative or positive numbers at the same time as an absolute value of 15 or more, it means that the flow has changed significantly. In this example, the flow rate of the syringe pump was changed from 0.3 to 0.0 ml/min.

Figure S3

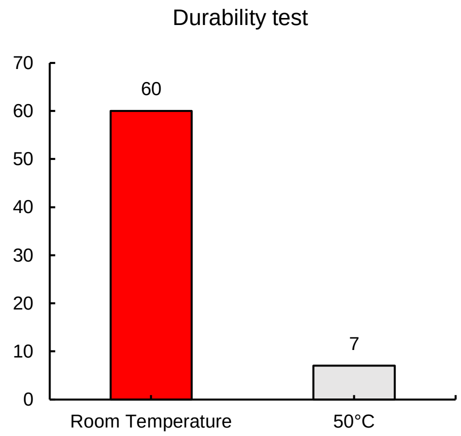

Figure S3: Characteristics of the durability of devices at room temperature and 50° C
